# Editorial: Multi-omics analysis of programmed cell death-mediated tumor microenvironment heterogeneity

**DOI:** 10.3389/fonc.2024.1265418

**Published:** 2024-03-26

**Authors:** Yanan Jiang

**Affiliations:** ^1^ Department of Pharmacology (State-Province Key Laboratories of Biomedicine-Pharmaceutics of China, Key Laboratory of Cardiovascular Research, Ministry of Education), College of Pharmacy, Harbin Medical University, Harbin, China; ^2^ Translational Medicine Research and Cooperation Center of Northern China, Heilongjiang Academy of Medical Sciences, Harbin, China

**Keywords:** programmed cell death, multi-omics, precise treatment, prognosis biomarker, microenvironment heterogeneity

## Introduction

Multiple programmed cell deaths (apoptosis, necroptosis, pyroptosis, ferroptosis, etc) play important roles in tumor initiation and progression (1). These cell death patterns have unique characteristics. For example, pyroptosis is an inflammation associated programmed cell death mediated by NLRP3/Caspase-1/GSDMD, which is accompanied by secretion of the inflammatory cytokines such as interleukins (ILs). ILs are important tumor microenvironment components, which have pleiotropic effects on tumor progression (2, 3). Programmed cell death-mediated tumor microenvironment heterogeneity also shows a profound association with tumor therapeutic responsiveness (4, 5). IL-1β promotes tumor immune evasion thus is associated with the prognosis of gastric cancer (6). By blocking the interleukin-1 (IL-1) pathway, resistance to immunotherapies can be overcome (7). The application of multi-omics data provides a comprehensive approach for cancer research and clinical treatment (8). A better understanding of programmed cell death-mediated tumor microenvironment heterogeneity based on multi-omics data will contribute to the cancer subtyping and individualized treatment.

The aim of the proposed Research Topic “*Multi-Omics Analysis of Programmed Cell Death-Mediated Tumor Microenvironment Heterogeneity*” is to provide new insights into the application of multi-omics data in the investigation of programmed cell death-mediated tumor microenvironment heterogeneity.

## Overview of the articles included in this Research Topic

Identification of effective biomarkers is important for the precise treatment of cancer. Chemotherapy and immunotherapy are the most widely used treatment in cancer. Drug resistance is a major obstacle limiting the clinical use of chemotherapy, such as 5-fluorouracil (5-FU) (9). Liu et al. systematically investigated the expression patterns and biological features of Smith-like (LSM) family members in gastric cancer and identified LSM5 and LSM8 as potential biomarkers for 5-FU-resistant gastric cancer. Similarly, LSM family members were also defined as novel unfavorable biomarkers for hepatocellular carcinoma. A score with four LSM family genes (LSM5, LSM10, LSM12, and LSM14B) could predict the overall survival of HCC patients (10). These studies provide a new LSM family-related perspective for tumor treatment. The application of immune checkpoint inhibitors (ICIs) has revolutionized cancer treatment. Due to the heterogeneity of tumor, the efficiency of ICI treatment was different among patients. Based on the panel sequencing data, Long et al. constructed a genetic mutation-based signature to predict the benefit of patients receiving ICIs (5). Pan et al. further screened the suitable classifier for melanoma patients based on the genetic mutation features from whole exome sequencing and clinical data of patients with melanoma. They then constructed a durable clinical benefit (DCB) model that could effectively predict the sensitivity of patients to ICIs. The construction of prediction models may be helpful for clinical decision-making and ameliorating the therapeutic effectiveness for patients.

Drug combination therapy is a widely used cancer treatment strategy. AZD4547 is a selective inhibitor of FGFR, which is preferable for tumor with deregulated FGFR. Ma et al. further explored the effect of AZD4547 on non-small cell lung cancer (NSCLC) cells without deregulated FGFR. They found that AZD4547 has a weak inhibitory effect on NSCLC cells that do not have deregulated FGFR, yet it potentiates the effect of nab-paclitaxel (11). Furthermore, FGFR inhibition can enhance the efficiency of nab-paclitaxel in gastric cancer models (12). Therefore, the combination of FGFR inhibitors with nab-paclitaxel offers a promising new approach in tumor treatment.

Plenty of studies revealed the association between cancer and programmed cell deaths. Zhang et al. analyzed the research status of cutaneous squamous cell carcinoma (CSCC) and programmed cell deaths. They found that the number of relevant publications was increased with the years. Immunotherapy is considered as a therapeutic breakthrough for advanced CSCC. Besides, more CSCC-related studies were focused on programmed cell death. Substantive researches targeting the programmed cell deaths may provide a promising approach for the treatment of CSCC.

Two review papers were collected in this topic. Yang et al. summarized the role of the endosomal sorting complex required for transport (ESCRT) in the repair of damaged plasma membranes during various programed cell deaths. The current findings indicated that ESCRT is a potential target to overcome drug resistance during tumor therapy. Plenty of studies have confirmed that Hippo signaling pathway is a key regulator of cancer. Xiang et al. reviewed the current findings of Hippo pathway in ferroptosis. They purposed that targeting ferroptosis would be a new therapeutic strategy for certain types of cancer. These findings emphasized the importance of programmed cell death in tumor progression and treatment.

## Conclusion

In summary, articles in this Research Topic highlighted the importance of programmed cell death in cancer. An in-depth understanding of the role of programmed cell death in tumor microenvironment heterogeneity based on multi-omics data will bring new insights into cancer research. Further studies on the identification of new biomarkers and therapeutic targets associated with programmed cell death in cancer are still necessary. We hope that this topic will continue to contribute to the study focused on this field.

## Author contributions

YJ: Writing – original draft, Writing – review & editing.
